# Enhancing infection control practices and biosecurity plans on Swedish pig farms: insights, challenges, and strategies

**DOI:** 10.1186/s13028-024-00771-9

**Published:** 2024-09-11

**Authors:** Elisabeth Rajala, Hedvig Gröndal, Johan Eriksson, Susanna Sternberg Lewerin

**Affiliations:** 1 Department of Animal Biosciences, Division of Bacteriology and Food Safety, Swedish University of Agricultural Sciences, P.O. Box 7054, 750 07 Uppsala, Sweden; 2 Sveriges Grisföretagare, Klockartorpsgatan 14, 723 44 Västerås, Sweden

**Keywords:** ASF, Biosecurity, Control, Pig production, Wild boar

## Abstract

**Background:**

African swine fever (ASF) poses a threat to the global pig industry, leading to significant economic losses and widespread disruptions in pig farming and associated sectors. In September 2023, the first case of ASF in Swedish wild boar triggered immediate responses from authorities, including the establishment of restricted zones and culling measures. A new ASF certification programme for pig herds was initiated to improve biosecurity and proactive disease management. This survey aimed to assess the sentiments and actions of Swedish pig farmers six months post-outbreak, particularly regarding biosecurity measures. Such information is important to improve preparedness for future disease threats. A questionnaire was distributed to members of the Swedish pig producers' organisation.

**Results:**

A total of 113 farmers responded (response rate 27%), with the majority considering the risk of ASF reappearing in Sweden as high. The estimated cost for connecting the farms to the ASF certification programme varied greatly, with a majority identifying cost as a substantial hurdle. While many farmers sought biosecurity advice from veterinarians, 43% had not implemented suggested measures. Over one third had not received concrete measures that would fit their farms, and 14% had not received any biosecurity advice from veterinarians at all. Discussions among farmers emphasized concerns about ASF outbreaks, transmission mechanisms, and regulatory compliance, highlighting the importance of ongoing communication and knowledge exchange to address the challenges posed by ASF effectively. Additionally, participants also mentioned the role of dense wild boar populations and shortcomings in municipal food waste management as important risk factors.

**Conclusions:**

The responding farmers expressed widespread concern about new ASF outbreaks. A majority identified cost as a substantial hurdle for joining the ASF certification programme. While many farmers consulted veterinarians for advice on biosecurity, a significant number had yet to implement suggested measures and one third had not received specific guidance suitable for their farms. Stakeholder conversations highlighted concerns about ASF outbreaks, transmission, and compliance. They also discussed the role of dense wild boar populations and issues with municipal food waste management as significant risk factors for ASF.

## Background

**Fig. 1:**
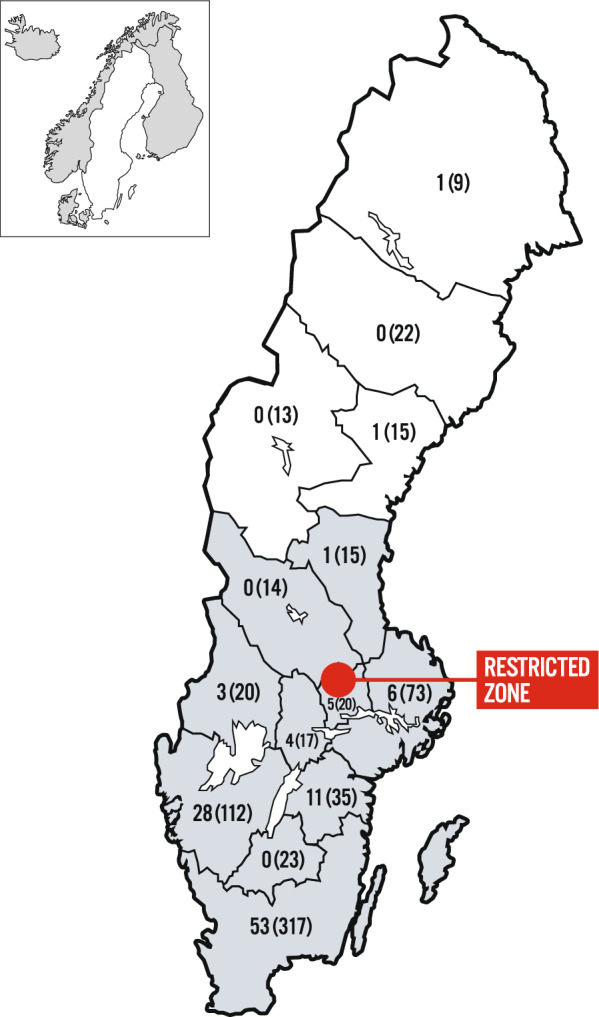
The geographical distribution of the respondents. The numbers represent the total number of respondents in each region, with the total number of pig producers in the same region in brackets. The colouring represents the presence of wild boar, with grey indicating their presence and white indicating their absence. The counties of Halland, Gotland, Kronoberg, Kalmar, Skåne, and Blekinge have been combined into a single region, as have the counties of Uppsala, Södermanland, and Stockholm

African swine fever (ASF) is a highly lethal viral disease affecting domestic pigs and Eurasian wild boar [1]**.** ASF poses a significant threat to the pig industry, causing substantial economic losses and disrupting pig farming and related industries in affected regions. Since its detection in Georgia in 2007, ASF has rapidly spread throughout Europe, impacting numerous countries [2]. ASF transmission in Europe primarily occurs through direct or indirect contact with infected wild boar or domestic pigs, or via ingestion of contaminated materials [3, 4]. Surveillance for ASF in wild boar in Sweden is mainly based on reports and examination of carcasses found [5]. Surveillance for ASF in domestic pigs is based on clinical/passive surveillance (i.e. owners reacting to symptoms or increased mortality and contacting a veterinarian). This surveillance strategy has been chosen as infection is associated with up to 100% lethality in domestic pigs as well as wild boar [6]. On 6 September 2023, the first case of ASF was confirmed in Swedish wild boar, all cases were found within a specific area, indicating a point-source introduction [7]. Around the affected area, an infected zone with restrictions according to Commission Implementing Regulation (EU) 2023/594 of one thousand square kilometres was established, where all activities, including forestry, agriculture, and hunting were banned, except for travel on main roads (Fig. 1). All pigs living in the infected zone were culled, which was 59 pigs from six farms in total [8]. The outbreak was subsequently eradicated by active search for wild boar carcasses, sampling and destruction of these, fencing off the infected area and culling all remaining wild boar within this area [9]. In the most optimistic scenario, Sweden could be declared free from ASF during the autumn 2024, one year after the outbreak [10].

In October 2023, the Swedish Board of Agriculture approved a new infection control certification programme for pig herds called "SSB ASF" with requirements based on relevant EU legislation [11]. To participate in the new programme, farms must meet not only standard internal and external biosecurity practices but also additional requirements. These include installing animal fencing around pig housing and areas where feed and bedding are stored, conducting an annual review of infection control procedures with an authorized veterinarian, implementing infection control measures during construction or repairs, and enforcing a 48-h access ban to the farm following hunting activities in restricted areas. Affiliation to the new ASF certification programme is voluntary and since December 2023 it has been possible for Swedish pig producers to sign up for the programme. The objectives of the new programme are to be proactive and to work with new issues concerning infection control and biosecurity. If an infection is detected in an area, it will be easier and quicker for farms connected to the programme to get authorisation to move their pigs. Herds that are not affiliated to the programme will instead have to implement the same biosecurity routines and put up fences immediately and then be checked by an official veterinarian to receive an approval from the Swedish Board of Agriculture to transport pigs to or from the herd.

Farmers play a crucial part in controlling contagious animal diseases, and their role in managing disease risks has been extensively studied across various livestock sectors, including pigs [12–14], poultry [15], and cattle [16, 17]. Pig keepers in Sweden have been advised to review their biosecurity measures and reminded to contact a veterinarian if there are signs of disease or increased mortality among the pigs [7]. The authors of the current paper conducted a previous survey targeting Swedish pig farmers in 2023, where the responding farmers in general were satisfied with the information received from authorities and other actors in the beginning of the ASF outbreak [14]. Moreover, the majority of the participants expressed optimism about the future, and significant emphasis was placed on implementing measures to manage the wild boar population as a crucial step in safeguarding Sweden's pig production.

The primary aim of this survey was to assess the current sentiments and actions of farmers six months post-outbreak, particularly regarding the implementation of suggested biosecurity measures. This study seeks to achieve two specific objectives, (i) evaluate the current state of infection control and biosecurity plans on pig farms in Sweden, and (ii) identify any barriers, whether financial or otherwise, that may prevent pig producers from executing crucial biosecurity measures effectively. Such information is important to improve preparedness for future disease threats, and to identify strategies to overcome barriers to biosecurity implementation.

## Methods

### Study population and study procedure

The study population and study procedure were the same as for the previous study and have been described in detail [14]. In brief, the study population was Swedish pig farmers who are members of the Swedish pig producers’ organisation Sveriges Grisföretagare [18]. The survey was distributed to approximately 430 pig farmers with the aim to enrol as many farmers as possible, but with no specific numerical target. A questionnaire was designed in Netigate (Netigate AB, Stockholm, Sweden) with approximately 20 questions on the participants’ risk perception and future outlook, ambition for biosecurity, costs for affiliation to the new ASF certification programme, and current biosecurity practices. The participants were informed that completing the questionnaire would take no more than 10–15 min, and that responses were anonymous, and participation was voluntary. The questions included a mix of open-ended, multiple-choice, and single-choice formats, with some presented using a Likert scale. Supplementary material S1 includes a translated version of the questionnaire. The questionnaire was distributed by the pig producers’ organisation to all their members via email on 12 March 2024, a reminder was sent out on 4 April, and the survey was closed after one month on 11 April 2024. The data were entered, cleaned, and analysed using descriptive statistics in Microsoft Excel.

## Results

### Participants

A total of 115 farmers responded to the survey (response rate 27%). Two of the respondents did not own pigs and were therefore removed from the analysis, resulting in 113 respondents. The geographical distribution of the farms is illustrated in Fig. 1. The majority (88.5%) of the 113 respondents kept their pigs strictly indoors, while 2.7% (n = 3) kept their pigs both indoors and outdoors. Additionally, 2.6% (n = 3) kept the pigs only outdoors, and 6.2% (n = 7) kept the pigs indoors with some outdoor access which means that the pigs have access to both indoor and outdoor areas. These are groups of pigs that are housed in a well-ventilated barn with large doors or sliding wall sections that can be opened during suitable weather conditions while the animals remain inside. Even though these pigs are still considered to be kept indoors, these more open barns present an opportunity for direct contact with for example wild boar, should they approach the building. In total, 76 respondents kept sows, 92 respondents kept slaughter pigs, and 60 respondents kept both sows and slaughter pigs. The median number of sows was 300 (range 20—1500), and the median herd size for slaughter pigs was 1600 (range 80—14500).

### Risk perception and future outlook

The majority of the 113 respondents (65%, n = 73) rated the risk of ASF reappearing in Sweden while still being an active pig producer as either 4 or 5, on a scale where 1 indicates no risk and 5 indicates a very high risk (Table 1). On the other hand, 62% (n = 71) rated the risk for their own herd becoming infected with ASF while being an active producer as either 1 or 2.

**Table 1:** Questions linked to Swedish pig farmers’ risk perception for African swine fever in 2024

Question	Category	n	%
How do you perceive the risk of ASF reappearing in Sweden while you are an active pig producer?^1^ (n = 113)	1 (no risk)	0	0
	2	11	10
	3	29	26
	4	40	35
	5 (very high risk)	33	29
How do you perceive the risk of your herd becoming infected with ASF during your time as an active producer?^1^ (n = 113)	1 (no risk)	5	4
	2	66	58
	3	30	27
	4	8	7
	5 (very high risk)	4	4

^1^Likert-scale question where 1 is equal to no risk, and 5 is equal to very high risk

In total, 78 farmers responded to the open question “How do you see the future of your pig production today?” where 52% (n = 41) expressed a positive outlook on the future. Twenty-four respondents (30%) expressed neither a positive nor a negative outlook on the future, and 9% (n = 7) indicated that either their production was constrained temporally or they harboured pessimistic sentiments about the future.

### Ambition for biosecurity

Very few of the respondents (5%, n = 6) had already signed up to the new ASF certification programme. Thirteen percent (n = 15) answered that they will join during 2024, and 19% (n = 22) that they need more time to prepare and will join in 2025. A few respondents (6%, n = 7) answered that they did not plan to join, and 34% (n = 39) that they will join if they end up in an ASF-restricted zone and it will be a requirement for transporting pigs to or from the herd. Of those who needed more information in order to make a decision (11%, n = 13) some requested more information about the costs, and more detailed information about farm specific biosecurity measures. A few respondents stated other alternatives such as they had not made a decision yet or that they were hesitant (3%, n = 3), that they will cease the production if their pigs get ASF (4%, n = 4), or that they did not think it was practically possible to fulfil the requirements for affiliation to the new ASF certification programme (2%, n = 2). Of those that did not plan to sign up to the programme, 46% (n = 17) stated that it is too expensive and that they would need financial support, 19% (n = 7) that the risk of ending up in an infected zone is small, and 19% (n = 7) that they will cease the production if they end up in an infected zone.

### Cost for affiliation to the new ASF certification programme

In total, 103 farmers estimated how much it would cost to sign up to the new programme (Table 2). The costs varied greatly between the farms. A follow-up question was if they would need financial support to achieve ASF certification for their herd, and if so, how much financial support they would need. Of 72 respondents in total, 54% (n = 39) provided a specific estimate of how much compensation that they would need (Table 2). Furthermore, 18% (n = 13) respondents stated that they would need 80–100% compensation, and 11% (n = 8) that they would need 50–75%. In addition, six of the respondents expressed that the farmers should not be forced to cover costs incurred by the irresponsible management of wild boar in the country, and one of them stated *“Why should we farmers pay all the time when we are innocent”*?

**Table 2:** Estimated costs for Swedish pig producers to connect to the new ASF certification programme, 2024

Question	Category	n	%
What is the estimated cost for affiliating your herd to the new ASF certification programme (SEK)? (n = 103)	< 100 000	7	7
	> 100 000–300 000	23	22
	> 300 000 – 500 000	22	21
	> 500 000 – 700 000	17	17
	> 700 000 – 1 million	8	8
	> 1 million	14	14
	Don´t know	12	12
If you would need financial support to sign up to the new programme, how much financial support would you need in SEK? (n = 72)	≤ 100 000	4	6
	> 100 000 – 300 000	14	19
	> 300 000 – 500 000	9	13
	> 500 000 – 700 000	5	7
	> 700 000 – 1 million	6	8
	> 1 million	1	1
	80—100% of the total cost	13	18
	50–75%	8	11
	Don´t know	12	17

### Current biosecurity measures

A majority of the 112 respondents (85%) had, since the first outbreak of ASF in September 2023, met with their farm veterinarian to discuss biosecurity measures (Table 3). Of those, 51% (n = 57) stated that they had received concrete and feasible measures, whereas 34% (n = 38) stated that they had not received any concrete measures that would suit their farm. Of those farmers that stated that they had not met the veterinarian to discuss biosecurity measures, but that they would like to do so (n = 12), three answered that they live in an area where there are no wild boars, two farmers stated that they already have good biosecurity measures in place, two farmers that they had no time to meet with the veterinarian, and one farmer stated that the veterinarian did not have time.

**Table 3:** Questions linked to biosecurity measures on pig farms answered by Swedish pig producers in 2024

Question	Category	n	%
Since the ASF outbreak in Fagersta, have you discussed specific biosecurity measures to protect your farm from infection? (n = 112)	Yes, we have had a briefing and received concrete and feasible measures	57	51
	Yes, we have had a briefing but have not received any concrete measures that would fit my farm	38	34
	No, we have not requested a meeting with our veterinarian	5	4
	No, we have not had a meeting but would like to	12	11
Will you implement the biosecurity measures proposed by your veterinarian? (n = 110)	I have not received any advice	15	14
	Yes, we have already implemented all proposed measures	8	7
	Yes, we have partly implemented all proposed measures	40	37
	We have not started yet but plan to implement all measures	33	30
	No, we have not implemented the proposed measures	14	13

A follow-up question was “Will you implement the biosecurity measures proposed by the veterinarian”. Of the 110 respondents, 44% (n = 48) stated that they had fully or partly implemented all proposed biosecurity measures, whereas 30% (n = 33) stated that they had not started yet but plan to implement all measures (Table 3). Of those respondents that stated that they had not implemented the proposed measures, some specified that there are no easy solutions (n = 2), or that the proposed measures are not feasible (n = 2).

The final question connected to biosecurity was “Do you discuss the risk of an ASF outbreak and the consequences of an outbreak with colleagues or others?” Of the 110 respondents in total, 67% (n = 74) answered “yes with other pig producers”, 59% (n = 65) responded “yes with my veterinarian”, 19% (n = 21) answered “yes but with other people”, and 11% (n = 12) answered “no I do not discuss with others”. Of those who answered that they discussed with other people they mentioned friends, neighbours, employees, hunters, landowners, consumers, politicians, and authorities. The subjects that they discussed were issues connected to biosecurity and how to implement the new regulations (n = 29). One of those respondents expressed “*How can you practically implement biosecurity measures on the farm to get a 100% protection and not just 95%? Can for example birds and flies carry the infection into the clean zone on the farm, or can the infection transmit with feed? If so, how do you protect yourself from it?*” Another topic the respondents discussed was economy, such as how to afford the implementation of new biosecurity measures (n = 2), the risk for ASF infection in the farm (n = 12), wild boar and the importance of reducing the population (n = 9), consequences for their specific farm and the society as a whole in case of a new ASF outbreak (n = 7), and municipalities’ waste management (n = 2). One respondent questioned how people and authorities in Sweden could be so naive as to allow professionals, such as foreign drivers, to bring food from other countries to Sweden and impose a significant risk of ASF virus introduction.

### Other comments

The last part of the survey gave the respondents the possibility to give free comments, and 18 persons responded. Four farmers emphasized the importance of reducing the wild boar population and one of them thought it was irresponsible of Swedish authorities to allow wild boar to spread uncontrolled. Another topic that was addressed was waste management and an incomprehension of how Swedish municipalities could be allowed to manage food waste so irresponsibly, posing a high risk of disease transmission. One respondent stated “*I could never imagine that municipalities such as Fagersta handled food waste in such a poor way, with wild boar rummaging through mountains of rubbish it is like playing Russian roulette with the entire Swedish pig industry*”. Along the same line, one respondent expressed that fast food restaurants along the roads where many leftovers are thrown away should be forced to use meat from the same country where the restaurant is located to mitigate the risk of disease transmission.

## Discussion

This survey shows that responding farmers are concerned about new ASF outbreaks. In addition, many respondents had not yet implemented recommended biosecurity measures and cost was identified as a major barrier to improving biosecurity.

The response rate for the current survey was 27%, which is approximately 10% of the total number of Swedish pig producers [19], and 19% of the members of Sveriges Grisföretagare [18]. This is a lower number compared to the previous survey in 2023 (response rate of 36%) [14]. There are no wild boars in the northern part of Sweden, but they are present in all counties from Värmland, Dalarna, and Gävleborg southwards [20] (Fig. 1). Only two of the respondents in the current survey were from areas with no wild boar population. One reason may be that farmers perceived the situation immediately after the first outbreak as very serious and were therefore more inclined to respond to the survey. However, the majority of the respondents in the current survey considered the risk of ASF reappearing in Sweden while still being an active pig producer as high. On the other hand, they were not so worried about their own farm being infected. Similar findings have been presented in Germany where most pig farmers did not perceive an increased threat to their farms despite the steady spread of ASF into and within the country [21]. Differences in risk perception in the current survey could be due to a combination of factors such as trust in their own practices and biosecurity measures to prevent infection on their farm, while recognizing the broader systemic challenges that could lead to ASF reintroduction into the country. Another reason could be differences in geographical locations of the farms and their proximity to the potential source of infection, such as a dense wild boar population, which is recognized as an important risk factor for disease transmission [14, 22].

Approximately half of the respondents expressed a positive outlook on the future, which is a small decline compared to the previous survey in 2023 by the same authors [14]. There may be several factors behind this such as increased costs or regulatory changes, but more research is needed to draw further conclusions.

Very few respondents had connected their herd to the new ASF certification programme, which was expected as the programme was launched in December 2023 [11]. Information about the new certification programme has been distributed to all pig producers in Sweden through various communication channels so the majority of the pig producers should be aware of the programme and what it contains. However, one third of the respondents in the current survey plan to sign up in 2024 or 2025. Previous research conducted in Germany has found that perceived strategy efficacy is the strongest direct predictor of the adoption of animal disease management strategies [23]. The willingness among the respondents in the current survey to improve their farm biosecurity might imply that they trust that the new biosecurity regulations will reduce the risk of infection. However, one third stated that they will only join the programme if they end up in an ASF-restricted zone, which means that it would be a requirement for them in order to transport pigs to or from the herd.

The respondents’ estimated cost for connecting their farms to the new ASF certification programme varied greatly, and more than 40% estimated that it will cost between 100 and 500 kSEK. Furthermore, 25% estimated the cost to be between 500 000 and 1 million SEK, and 14% more than 1 million SEK. A majority of the respondents would also need financial support to connect to the ASF certification programme. These results highlight the importance of considering financial support mechanisms and tailored assistance programs to facilitate the successful implementation of the ASF programme and ensure broader participation.

A majority of the respondents had, since the first outbreak of ASF in September 2023, consulted their farm veterinarian about biosecurity measures. Half of them had received concrete and feasible measures, whereas more than one third stated that they had not received any concrete measures that would suit their farm. This highlights the importance of a close cooperation between pig farmers and veterinarians, and a focus on joint decision-making, taking into account the individual circumstances of the farmers which has been acknowledged in other studies [21].

A notable finding was that 43% of farmers had not put into practice the biosecurity measures recommended by their veterinarian. However, 30% of them expressed intentions to do so in the future. Additionally, a concerning 14% of the respondents reported not having received any guidance from their veterinarian at all. Previous research with Swedish pig farmers and pig veterinarians indicates that their communication about biosecurity could be improved for better implementation [24]. These findings demonstrate the importance of collaboration between farmer and veterinarian, and a focus on the specific needs and objectives of the individual farmer. In a more positive light, more than forty percent of the respondents had fully or partially implemented all suggested biosecurity measures, which implies that they trust the advice of veterinarians and that the recommendations were affordable and feasible. Along the same line, the majority of the respondents tend to discuss the looming threat of ASF with colleagues and veterinarians. These conversations centre on concerns about new outbreaks, uncertainties about how ASF spreads, best practices for farm protection, and compliance with new regulations. Additionally, participants also touch on related issues like the role of dense wild boar populations in disease transmission and shortcomings in municipal food waste management. The latter reflects current theories on how ASF was introduced to Sweden through contaminated pork originating from a country (in e.g. Europe or Asia) affected by ASF genotype 2 that ended up in the garbage dump or in the surrounding environment within reach of wild boar [10]. This aligns with earlier studies where wild boar have been identified as the primary concern for disease spread in Sweden [14, 22]. The findings of the current survey underscore the critical need for continuous communication and knowledge exchange among stakeholders such as farmers, authorities, and veterinarians to effectively address the challenges posed by the disease and its consequences.

Potential bias could arise from the survey sample being drawn from farmers who voluntarily participate, potentially leading to underrepresentation of farmers who are less engaged or have different perspectives, potentially skewing the results towards those viewpoints. Efforts have been made to distribute the survey widely to mitigate this bias, and the farm demographics in the current survey align with national levels. Therefore, we consider the respondents to be representative of the target population. Also, respondents may provide socially desirable responses or answers that they believe align with the perceived expectations of the researchers. Measures such as anonymity and confidentiality have been implemented to encourage candid responses.

## Conclusions

In conclusion, this study provides insights into the current sentiments and actions of Swedish pig farmers six months post-outbreak of ASF. While the majority of respondents perceived the risk of ASF reappearing in Sweden as high, they express less concern about the risk of their own herd becoming infected. The majority of the respondents had consulted their farm veterinarian about biosecurity measures since the ASF outbreak, but many did not perceive that the measures would suit their farm, highlighting the importance of a close cooperation between pig farmers and veterinarians. The estimated cost for joining the new ASF programme varied greatly, with a majority identifying cost as a substantial hurdle. Discussions among respondents, their colleagues, and other stakeholders reflect the need for ongoing communication and knowledge exchange to effectively address the challenges posed by ASF.

## Data Availability

All data are available from the corresponding author upon reasonable request.
